# Uptake of Dicyanogold(I) by H9 Cells

**DOI:** 10.1155/MBD.1994.521

**Published:** 1994

**Authors:** Pamela W. Roy, R. C. Elder, Katherine Tepperman

**Affiliations:** 1 Department of Biological Sciences and Chemistry Biomedical Chemistry Research Center University of Cincinnati Cincinnati OH 45221-006 USA


### UPTAKE OF DICYANOGOLD(I) BY H9 CELLS

Pamela W. Roy, R. C. Elder and Katherine Tepperman

Department of Biological Sciences and Chemistry, Biomedical Chemistry Research Center, University of Cincinnati, Cincinnati, OH 45221-006, USA

Our analyses of blood and urine samples from patients treated with gold-based antiarthritis drugs have shown that the dicyanogold(I) anion, $[Au(CN)_2]^-$, is a common metabolite. Previous work by Graham had suggested that $[Au(CN)_2]^-$ would form as a result of interaction of any of the gold-based drugs with cyanide and that $[Au(CN)_2]^-$ would be readily taken up by cells. Our own work with red blood cells indicates that in vitro incubation with $[Au(CN)_2]^-$ leads to rapid uptake of gold.

Several reports have suggested that gold compounds may inhibit the production of the AIDS virus, HIV. Blough, at the Second International Conference on Gold and Silver in Medicine, showed that gold(I) thiolates, such as gold(I)thioglucose, can inhibit the activity of the HIV reverse transcriptase in vitro. However, gold(I)thioglucose does not enter cells readily, and thus the intracellular concentrations sufficient for inhibition of the viral reverse transcriptase cannot be achieved. We propose that $[Au(CN)_2]^-$ because of its ability to readily promote the uptake of gold by cells may be useful for the inhibition of HIV replication. To examine this, we have begun studies using a cell culture model system, H9 cells. These are a continuous line of $CD4^+$ T-lymphocytes, which support the growth of HIV in vitro.

Initially, H9 cells were incubated with varying concentrations of $[Au(CN)_2]^-$. External concentrations of 10 or 20 ppb had no effect on the growth rate of the cells. Incubation in 50 ppb $[Au(CN)_2]^-$ resulted in an initial decrease in growth rate, but by day three of culture, the doubling time for the cells had returned to control rates. Incubation with 100 ppb $[Au(CN)_2]^-$ resulted in a significant loss of cells, but again, by day three the rate of replication of the cells approaches normal levels. These results are in contrast to incubations of the H9 cells with Myochrysine. Concentrations of Myochrysine up to 0.1 mM (20 ppm) had little effect on growth rate of the cells.

We have used flow injection analysis (FIA) with inductively coupled plasma-mass spectrometric detection (ICP-MS) to monitor the amount of gold taken up by H9 cells when incubated with $[Au(CN)_2]^-$ for one hour at 37$^{o}$. The amount of gold taken up by the cells is dependent on the external concentrations. Cytosol concentrations are at least 70 fold higher than the concentration of gold in the external medium. Thus, $[Au(CN)_2]^-$ is taken up readily by the H9 cells and the intracellular level of gold is significantly higher than that in the external medium.

The mechanism of uptake of gold as a result of incubation with $[Au(CN)_2]^-$ is not known. One model for its uptake is the so called "sulfhydryl shuttle", proposed for other gold complexes, in which the gold interacts with surface sulfhydryls. Then, through a series of exchange reactions, the gold moves through the cell membrane. To test this model, we have treated the H9 cells with N-ethylmaleimide (NEM), then incubated the cells with $[Au(CN)_2]^-$. Since NEM blocks surface sulfhydryls, it should inhibit uptake through the sulfhydryl shuttle. Preincubation of the H9 cells with 1mM NEM for one half hour results in significant inhibition of the uptake of gold from $[Au(CN)_2]^-$. This result is consistent with uptake via the sulfhydryl shuttle. Further tests of the model are in progress.

